# Post-neoadjuvant treatment with capecitabine and trastuzumab emtansine in breast cancer patients—sequentially, or better simultaneously?

**DOI:** 10.1007/s00066-020-01667-z

**Published:** 2020-07-31

**Authors:** Marc D. Piroth, David Krug, Felix Sedlmayer, Marciana-Nona Duma, René Baumann, Wilfried Budach, Jürgen Dunst, Petra Feyer, Rainer Fietkau, Wulf Haase, Wolfgang Harms, Thomas Hehr, Rainer Souchon, Vratislav Strnad, Rolf Sauer, Breast Cancer Expert Panel of the German Society of Radiation Oncology (DEGRO)

**Affiliations:** 1 Dpt. of Radiation Oncology, Wuppertal University Hospital (Helios), Witten/Herdecke University, Heusnerstraße 40, 42283 Wuppertal, Germany; 2 University Hospital Schleswig-Holstein, Kiel, Germany; 3 Paracelsus Medical University Hospital Salzburg, Salzburg, Austria; 4 Friedrich-Schiller-University Hospital Jena, Jena, Germany; 5 St. Marien-Krankenhaus Siegen, Siegen, Germany; 6 Heinrich-Heine-University Hospital Düsseldorf, Düsseldorf, Germany; 7 Vivantes Hospital Neukoelln, Berlin, Germany; 8 University Hospital Erlangen, Erlangen, Germany; 9 formerly St.-Vincentius-Hospital Karlsruhe, Karlsruhe, Germany; 10 St. Claraspital Basel, Basel, Switzerland; 11 Marienhospital Stuttgart, Stuttgart, Germany; 12 formerly University Hospital, Tübingen, Germany

**Keywords:** Breast cancer, Radiotherapy, Radiochemotherapy, Capecitabine, T‑DM1

## Abstract

**Purpose:**

Following neoadjuvant chemotherapy for breast cancer, postoperative systemic therapy, also called post-neoadjuvant treatment, has been established in defined risk settings. We reviewed the evidence for sequencing of postoperative radiation and chemotherapy, with a focus on a capecitabine and trastuzumab emtansine (T-DM1)-based regimen.

**Methods:**

A systematic literature search using the PubMed/MEDLINE/Web of Science database was performed. We included prospective and retrospective reports published since 2015 and provided clinical data on toxicity and effectiveness.

**Results:**

Six studies were included, five of which investigated capecitabine-containing regimens. Of these, four were prospective investigations and one a retrospective matched comparative analysis. One randomized prospective trial was found for T‑DM1 and radiotherapy. In the majority of these reports, radiation-associated toxicities were not specifically addressed.

**Conclusion:**

Regarding oncologic outcome, the influence of sequencing radiation therapy with maintenance capecitabine chemotherapy in the post-neoadjuvant setting is unclear. Synchronous administration of capecitabine is feasible, but reports on possible excess toxicities are partially conflicting. Dose reduction of capecitabine should be considered, especially if normofractionated radiotherapy is used. In terms of tolerance, hypofractionated schedules seem to be superior in terms of toxicity in concurrent settings. T‑DM1 can safely be administered concurrently with radiotherapy.

Neoadjuvant chemotherapy (NACT) has been widely adopted into the multidisciplinary management of breast cancer and response to NACT correlates with the patient’s prognosis [1, 2].

Recently, it could be shown that in the case of pathological non-complete response (non-pCR) after NACT, post-neoadjuvant chemotherapy with capecitabine in patients with triple-negative disease (CREATE-X-trial [3]) and trastuzumab emtansine (T‑DM1) in patients with HER2-overexpressing tumors (KATHERINE-trial [4]) improves the prognosis.

Typically, patients with an indication for post-neoadjuvant treatment have a high risk for both locoregional and distant recurrence [1]. Thus, within the interdisciplinary tumor boards, the gynecological, medical, and radiation oncologist can argue well about the optimal treatment sequence. Using a sequential approach, either adjuvant radiotherapy or systemic therapy will be delayed by several weeks or even months. The simultaneous use may be the optimal sequence to avoid this problem. However, is it justifiable?

Concurrent radiochemotherapy is an approach that has been used for quite some time in patients with breast cancer. In the neoadjuvant setting, this mostly comprised patients with locally advanced or inflammatory breast cancer who were refractory to standard chemotherapy (recent overview by Corradini et al. [5]). In the adjuvant setting, randomized controlled trials of 5‑FU-containing regimens (along with cyclophosphamide and methotrexate or mitoxantrone) given concurrently vs. sequentially with adjuvant radiotherapy were conducted in the 1990s [6–10]. In these studies, an improvement in oncologic outcome, at least in subgroups, could be seen with concurrent administration of chemotherapy, with the exception of one trial [8]. Regarding side effects, the concurrent regime led to an increase in acute skin toxicities in all trials except one [9]. Also, late effects such as telangiectasia, fibrosis, and breast shrinkage were more common in the concurrent setting.

However, the results of these trials regarding toxicity and effectiveness can only partly be extrapolated to the concurrent use of capecitabine, a prodrug of (5-Fluorouracil (5‑FU), since the dosage of 5‑FU is not comparable and, additionally, mitoxantrone and cyclophosphamide were used.

In the present review, we therefore tried to compile more evidence regarding the oncologic effectiveness as well as the toxicity and tolerability of concurrent versus sequential use of modern maintenance systemic medications, in particular capecitabine and trastuzumab or T‑DM1. Corresponding studies are shown in Table 1.

**Table 1:** Radiotherapy toxicity in dependency of capecitabine and trastuzumab/T-DM1 sequence

Author/study	Study type	*n* (total)	*n* (patients with RT)	Chemotherapy	Sequence chemotherapy/RT	RT fractionation	RT-associated toxicity
Masuda CREATE‑X [3]	RCT phase III	910	647	+/− Cap 6–8 cycles, 1250 mg/m^2^, 2 ×/d, days 1–14	Sequential	“standard”	Not specifically stated
Lluch GEICAM/CIBOMA [11]	RCT phase III	876	352	+/− Cap 8 cycles, 1000 mg/m^2^ 2 ×/d, days 1–14	Sequential (RT first)	Not reported	Not specifically stated
Alhanafy [14]	RCT	100	100 (PMRT)	+/− Cap 825 mg/m^2^ 2 ×/d, during RT days	Synchronous	Hypo-fractionated (40 Gy/15 Fx) (>60%/patients)	Early G1/2: 22% (Cap) vs 18% (control), n. s. late effects: no difference
Woodward [13]	Single-arm phase II	26	26	Cap 825 mg/m^2^ 2 ×/d, during RT days^a^	Synchronous	50–72 Gy (single dose 1.5–2.2 Gy), median 66 Gy	Not specifically stated
Liu [12]	Retrospective matched comp	78	21	Cap 1000–2000 mg/day during RT days	Synchronous	Standard (6–8 weeks)	No excess acute radiation toxicity
v. Minckwitz KATHERINE [4]	RCT phase III	1486	Not mentioned (>50%)	Trastuzumab +/− emtansine (T-DM1) 14 cycles	Synchronous	Not elaborated (following “institutional protocols”)	Any grade 27.6% (trastuzumab) vs 25.4% (T-DM1) n. s.; G3: 1% vs 1.4%, n. s.
Fernando SECRAB [10]	RCT phase III	2296	2296	6–8 CMF or anthracycline-CMF	1150 synchronous (between chemo-cycles); 1146 sequential (after chemo completion)	15 Fx (40 Gy) or >15 Fx (50 Gy/25 Fx or 45 Gy/20 Fx)	Moderate/severe acute skin tox: 24% (sync.) vs. 14.8% (seq.); moderate/severe telangiectasia: 3.0% (sync.) vs. 1.7% (seq.); acute skin reactions: 24.5% (>15 Fx) vs. 16.7% (15 Fx); teleangiectasia: >15 Fx vs. 15 Fx—no difference

*RCT* radiochemotherapy, *RT* radiochemotherapy, *PMRT* postmastectomy radiotherapy, *Cap* capecitabine, *CMF* cyclophosphamide, methotrexate, 5‑fluoruracil

^a^After 9 patients reduced after initial daily application (excess toxicity), *Fx* fractions

## Capecitabine and radiotherapy

### CREATE-X-trial

The Capecitabine for Residual Cancer as Adjuvant Therapy (CREATE-X) trial, enrolling patients in Japan and Korea with HER2-negative breast cancer and residual invasive disease after NACT, demonstrated an improvement in 5‑year overall survival (89.2% vs. 83.6%, HR 0.59, *p* = 0.01) by the addition of further adjuvant chemotherapy with capecitabine [3]. In subgroup analyses, this effect was only confirmed in patients with triple-negative breast cancer.

The trial protocol specified that adjuvant radiotherapy (RT) should be performed as per standard guidelines. Radiotherapy could be given before or after randomization and could be concomitant with postsurgical endocrine therapy. However, it did not specify RT schedules, including the order or timing of intervals between capecitabine and RT treatments. Nonetheless radiotherapy was—per protocol—not intended to be given simultaneously with capecitabine.

The capecitabine dose used in the CREATE-X-trial was 1250 mg/m^2^ twice per day on days 1 to 14, every 3 weeks for six or eight cycles. This dose is higher than usually used in simultaneous radiochemotherapy schedules, e.g. for rectal cancer (825 mg/m^2^ twice per day).

### GEICAM/2003_CIBOMA/2004 trial

The prognostic advantage using capecitabine shown by the CREATE-X-trial cannot be transferred to all triple-negative patients, as shown by the recently published GEICAM/2003_CIBOMA/2004 trial [11]. Within this trial, an extended adjuvant capecitabine regime after completion of standard chemotherapy and also completion of adjuvant radiotherapy was evaluated. Patients with triple-negative breast cancer and lymph node positivity and/or tumor ≥1 cm were included. In this recently published study, no significant improvement in disease-free survival (primary endpoint) could be seen.

### Concurrent use

In a retrospective study including 317 patients who received doxorubicin and taxan-based neoadjuvant chemotherapy from 2004 to 2016, Liu et al. identified 21 patients receiving adjuvant radiotherapy over 6–8 weeks and simultaneous capecitabine [12]. In all patients, the whole breast and also the lymphatic pathways were included into the radiation fields. The capecitabine schedules used in this small cohort were available in detail in 16 patients. The schedules were different, with 1000 mg (*n* = 5), 1500 mg (*n* = 3), and 2000 mg (*n* = 8). The drug was given 5 days per week in two divided daily doses. The authors found that the concurrent use of capecitabine with RT did not compromise compliance with RT and no significant increase in acute radiation toxicity was seen. According to this subgroup analysis, simultaneous use seems to be feasible. However, these findings should be interpreted with caution due to the small patient number.

Woodward et al. conducted a prospective phase II study of neoadjuvant/definitive concurrent radiochemotherapy with capecitabine in patients refractory to standard chemotherapy [13]. Overall, 32 patients were included between 2009 and 2012. Capecitabine was initially used with a dose of 825 mg/m^2^ twice daily for the whole duration of the radiotherapy course. After 10 patients the scheduling was changed due to excessive toxicity and capecitabine was administered only from Monday to Friday. Three different radiotherapy regimens were used, each administering a dose of at least 50 Gy with a median dose of 66 Gy. Grade 3 toxicities were mostly gastrointestinal (diarrhea, nausea, or vomiting) and/or hand-foot skin reactions, with 50% of patients developing grade ≥3 treatment-related dermatologic toxicity. However, the authors did not discriminate between skin reactions as part of hand-foot syndrome or excess reactions in conjunction with radiation fields (or others). Thus, local tolerability of concurrent radiation remains unclear.

The authors conclude that concurrent capecitabine can be safely administered using a non-continuous dosing regimen with careful clinical monitoring.

A prospective randomized study was conducted in Egypt and published in a non-PubMed ranked journal [14]. Alhanafy et al. evaluated the toxicity, feasibility, and efficacy of concurrent capecitabine with radiotherapy in high-risk breast cancer patients [14]. All patients had had mastectomy with axillary dissection and adjuvant chemotherapy previously. High-risk features were T3 or T4 tumor and/or ≥4 positive axillary lymph nodes. Overall, 100 patients were included within 8 months in 2011. Patients were randomized 1:1 to the radiotherapy only group (40 Gy in 15 fractions over 3 weeks with 2D simulator-based radiotherapy) or the radiotherapy and capecitabine (825 mg/m^2^, twice daily on all radiotherapy days) group. In nearly 90% of cases the supraclavicular region was included into the radiation volume. No treatment of the internal mammary nodes was performed.

No early grade III or IV toxicity was seen and the early grade I/II toxicities were comparable: 14%/4% and 18%/4% of patients had grade I/II radiation dermatitis; only one patient in each group had an acute radiation pneumonitis (grade I). Within a 2-year follow-up, no decrease of the left ventricular ejection fraction was seen. Also, no ischemic heart manifestation, symptomatic rib fracture, or plexopathy could be detected. Regarding the late effects in 2 patients (4%) in the concurrent arm and in 1 patient (2%) in the radiotherapy-only arm a grade III fibrosis/fat necrosis was seen. Regarding oncologic outcome, local and distant control were not statistically different. Though this is the only randomized trial, it has to be mentioned that the toxicity rates were low in the control arm compared to other randomized controlled trials [15, 16], which is presumably attributable to the hypofractionated radiotherapy.

According to these data, hypofractionated radiotherapy to 40 Gy in combination with simultaneous capecitabine does not increase toxicity.

In this context it is interesting to mention that in the SECRAB trial, which studied simultaneous vs. sequential use of 5‑FU-based chemotherapy in breast cancer patients, hypofractionation (radiotherapy with 15 fractions versus >15 fractions) was associated with lower acute side effects [10]. Therefore, Fernando et al. advised that patients treated with synchronous chemoradiotherapy should be treated with a three-weekly radiotherapy regimen to avoid additional acute skin toxicity [10].

## Capecitabine and 5FU

### Heart toxicities

The risk of cardiac toxicity during fluoropyrimidine-based treatment ranges from 1.2% to 18%, although larger trials have suggested an incidence of symptomatic cardiotoxicity of 1.2–4.3% during treatment [17]. A large meta-analysis on breast and colorectal cancer patients treated with fluoropyrimidines confirmed this observation, revealing a similar low incidence (3%) of symptomatic 5‑FU- and capecitabine-related events [18]. In a prospective study, symptomatic ECG abnormalities were significantly more frequent in patients treated with 24-hour infusion of 5‑FU and with capecitabine than in those receiving a short infusion of 5‑FU [19].

Often, in high-risk patients who will undergo post-neoadjuvant capecitabine, concomitant irradiation of the internal mammary region is indicated. For patients with left-sided breast cancer this could result in higher heart doses. A link between cardiotoxicity and previous chest radiotherapy or baseline renal function impairment has been suggested for 5‑FU or capecitabine. It remains unclear whether the combination of radiation therapy and capecitabine might result in different late heart toxicities [20].

Concerning this, the concurrent use of radiation therapy and capecitabine may not be recommended if the left-sided breast/chest wall including the internal mammary nodes are treated.

Recently published technical recommendations and cardiac dose constraints [21, 22] should be respected.

## Trastuzumab emtansine (T-DM1)

Besides capecitabine, another post-neoadjuvant treatment option was recently introduced for patients with HER2-positive breast cancer and residual invasive disease after NACT. In the KATHERINE-trial, led by the German Breast Group, patients were randomized to receive adjuvant T‑DM1 or trastuzumab for 14 cycles [4]. Trastuzumab and T‑DM1 were given concurrently with adjuvant radiotherapy, if indicated. T‑DM1 significantly improved invasive disease-free survival and freedom of distant recurrence, with a borderline improvement of overall survival. Toxicity in the T‑DM1 arm was significantly higher compared to the trastuzumab-arm. However, regarding radiotherapy-related skin injury and radiation-associated pneumonitis (1.5% vs. 0.7%), there were no significant differences between the treatment arms in the published data. Loibl et al. recently presented a subgroup analysis regarding adjuvant radiotherapy at the ESMO Breast Cancer Virtual Meeting 2020 [23]. In summary, there was no difference regarding the benefit of T‑DM1 according to the administration of adjuvant radiotherapy. There was an increase in grade ≥3 adverse events in the T‑DM1 arm in patients treated with adjuvant radiotherapy as compared to patients not receiving radiotherapy (27.4% vs. 16.4%) or patients treated with trastuzumab with or without radiotherapy (15.6% vs. 14.6%). Adverse events ≥3 occurring more often in the T‑DM1 arm in patients who received radiotherapy included radiation dermatitis (1.6% vs. 0%) and mastitis (0.8% vs. 0%). Regarding all-grade events in the T‑DM1 arm, pulmonary toxicity (3.4% vs. 0%) and cardiac toxicity (3.4% vs. 1.7%) were more frequent in patients who received radiotherapy and were mostly limited to low-grade toxicity. Cardiac events were more frequent in the trastuzumab arm. Overall, this is in accordance with the previously published safety profile of T‑DM1.

Regarding the concurrent use of T‑DM1, the same caution as for trastuzumab is advised in case of left-sided breast cancer and/or internal mammary node irradiation [24, 25].

The use of trastuzumab concurrently with radiotherapy is not associated with increased acute toxicity [26]. The prospective trials assessing this combination in breast cancer patients showed acceptable skin and esophageal toxicity and cardiac toxicity rates may be similar, regardless of whether trastuzumab is given concomitantly or sequentially [24].

## Conclusion

Regarding the use of capecitabine concurrently with radiotherapy in breast cancer patients, several factors have to be taken into account.

There is no certain oncologic benefit of concurrent versus sequential adjuvant radiochemotherapy. The benefit in the CREATE‑X trial was shown with 6–8 cycles of full-dose capecitabine and sequential radiotherapy. Concurrent administration of capecitabine with adjuvant radiotherapy is feasible, with a higher risk of acute toxicity. It is evident that hypofractionated radiotherapy may be advantageous due to the lower skin-related acute toxicity and the lower concomitant exposure. Dose reduction of capecitabine to 825 mg/m^2^ twice daily on radiotherapy days without chemotherapy administration at weekends should be considered, especially if normofractionated radiotherapy is used. It is not clear whether dose reduction during radiotherapy might impair the benefit seen in the CREATE‑X trial.

Alternatively, hypofractionated radiotherapy might also be given prior to adjuvant capecitabine with a delay of post-neoadjuvant treatment of only 3–4 weeks compared to 5–7 weeks with normofractionated radiotherapy.

The decision regarding scheduling and dose of capecitabine in patients with an indication for adjuvant radiotherapy should be made in the interdisciplinary tumor board. Concurrent radiochemotherapy may be used in patients with a high risk of locoregional recurrence after individual counseling of the patient regarding an increase in acute and potentially also late toxicity.

T‑DM1 can safely be administered concurrently with radiotherapy, as shown in the KATHERINE trial. Caution is advised regarding use in patients with left-sided irradiation and those with an indication for internal mammary node irradiation [21, 22].
